# Supplementary material for: The interactome of intact mitochondria by cross-linking mass spectrometry provides evidence for coexisting respiratory supercomplexes
Source: Mol Cell Proteomics. 2017 Dec 8;17(2):216–32. doi: 10.1074/mcp.RA117.000470 (PMC5795388; doi:10.1074/mcp.RA117.000470)
Supplement: Supplemental Data [file supp_RA117.000470_133922_0_supp_23978_fzffwf.zip › spectra_annotation/mito_spectra_annotation/66-1-8-1-8-1.pdf]

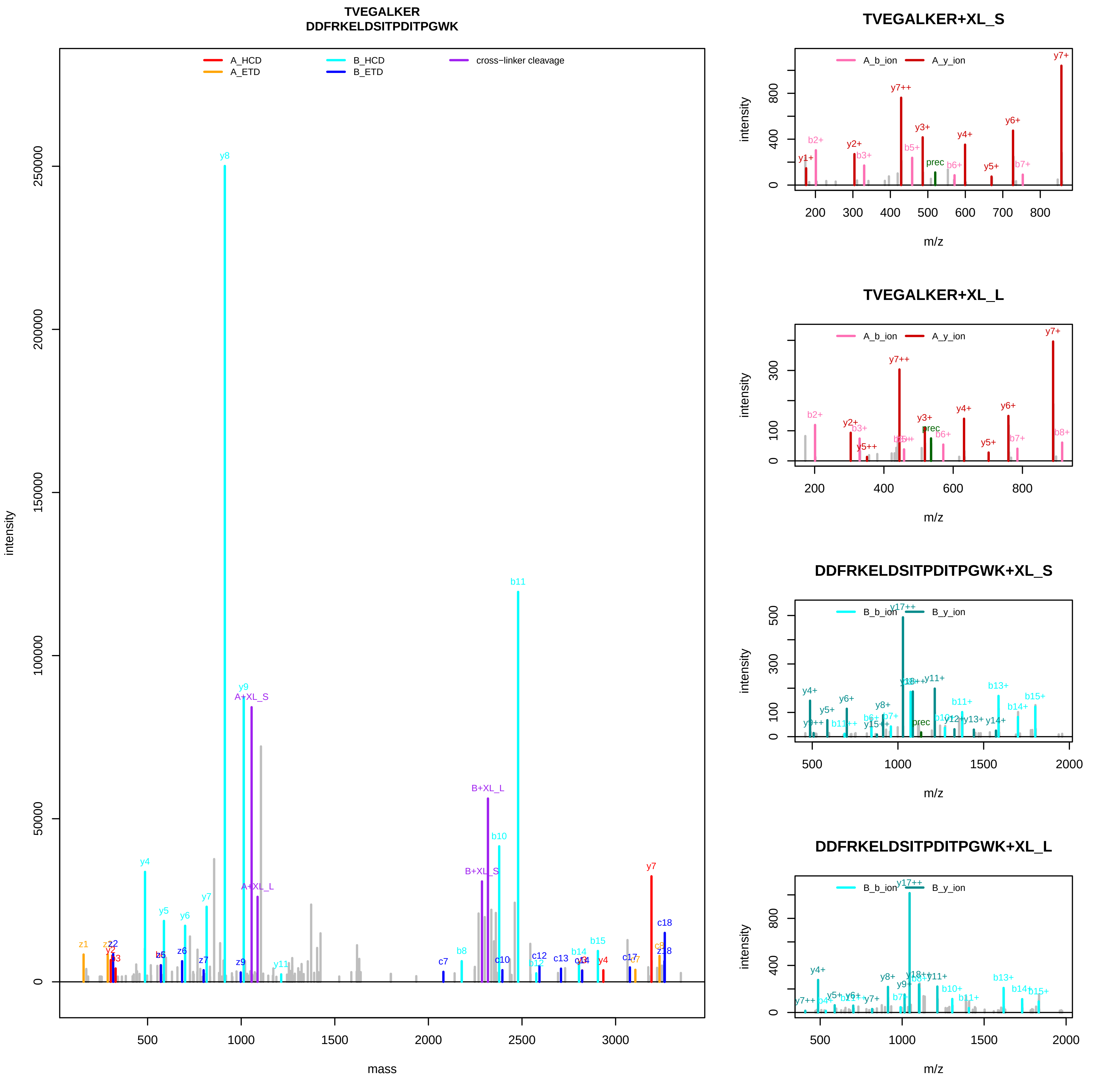

TVEGALKER
DDFRKELDSITPDITPGWK
A_HCD
A_ETD
B_HCD
B_ETD
cross−linker cleavage
intensity
mass
0
50000
100000
150000
200000
250000
500
1000
1500
2000
2500
3000
y8
b11
y9
A+XL_S
A+XL_L
B+XL_L
B+XL_S
b10
y7
y4
y5
y6
y11
b8
b14
b15
c7
c10
c12
c13
c17
c18
z18
z1
z2
z5
z6
z7
z9
y2
y4
TVEGALKER+XL_S
A_b_ion
A_y_ion
m/z
y1+
b2+
y2+
b3+
y7++
b5+
y3+
prec
b6+
y4+
y5+
y6+
b7+
y7+
200
300
400
500
600
700
800
0
400
800
TVEGALKER+XL_L
A_b_ion
A_y_ion
m/z
b2+
y2+
b3+
y5++
y7++
y3+
prec
b6+
y4+
y5+
y6+
b7+
y7+
b8+
200
400
600
800
0
100
300
DDFRKELDSITPDITPGWK+XL_S
B_b_ion
B_y_ion
m/z
y17++
y4+
y5+
y6+
y8+
y11+
b11+
b13+
b14+
b15+
y13+
y14+
prec
500
1000
1500
2000
0
100
300
500
DDFRKELDSITPDITPGWK+XL_L
B_b_ion
B_y_ion
m/z
y17++
y4+
y8+
y11+
b10+
b11+
b13+
b14+
b15+
y7+
y9+
500
1000
1500
2000
0
400
800
